# Protective effect of *Rheum turkestanicum* root against mercuric chloride-induced hepatorenal toxicity in rats 

**Published:** 2018

**Authors:** Azar Hosseini, Arezoo Rajabian, Sahar Fanoudi, Mahdi Farzadnia, Mohammad Taher Boroushaki

**Affiliations:** 1 *Pharmacological Research Center of Medicinal Plants, Mashhad University of Medical Sciences, Mashhad, Iran*; 2 *Department of Pharmacology, Faculty of Medicine, Mashhad University of Medical Sciences, Mashhad, Iran*; 3 *ancer Molecular Pathology Research Center, Imam Reza Hospital, Faculty of Medicine, Mashhad University of Medical Sciences, Mashhad, Iran*

**Keywords:** HgCl_2_, Oxidative stress, Rheum turkestanicum, Lipid peroxidation

## Abstract

**Objective:**

The present study was designed to investigate the protective effects of hydroalcoholic extract of *Rheum turkestanicum* against HgCl_2_ hepatorenal toxicity in rats.

**Materials and Methods:**

Animals were randomly divided into five groups (n= 6 in each group) and received HgCl_2_ and plant’s extract, intraperitoneally. Group1 received saline (1 mL/kg/day), group 2 received extract (200 mg/kg/day), group 3 was treated with HgCl_2_ (5 mg/kg/day,) and groups 4 and 5 received the extract (100 and 200 mg/kg/day, respectively), 1 hr before HgCl_2_ administration. All injections last for 3 days. Blood samples and specimens of the liver and kidney were collected 24 hr after the last injection.

**Results:**

Data showed that HgCl_2_ significantly increases liver malondialdehyde (MDA) level, reduces total sulfhydryl content and increases serum alanine aminotransferase (ALT) and aspartate aminotransferase (AST) activity, compared to control group. The histopathological changes such as inflammatory cells infiltration was observed in HgCl_2_-treated group while plant’s extract partially improved histological changes. The extract (100 and 200 mg/kg/day) improved the liver functions as reflected by significant reductions in AST and ALT levels in serum, MDA decreased and the content of total sulfhydryl elevated. Also, the extract improved necrosis and atrophy of the kidney induced byHgCl_2_. Pretreatment with the extract reduced creatinine and urea in serum, and glucose and protein concentrations in urine, compared to HgCl_2_- treated group (group III). The extract significantly reversed HgCl_2_-induced depletion in thiol content and elevation in MDA content.

**Conclusion:**

Therefore, oxidative stress may play an important role in HgCl_2_-induced hepatorenal injury and *R. turkestanicum* extract may be regarded as a useful to protect the kidney and liver against HgCl_2_-induced oxidative damage.

## Introduction

Mercury is an environmental and industrial pollutant. It induces severe alterations in tissues of both humans and animals (Mahboob et al., 2001 ▶; Şener et al., 2003 ▶). Different forms of mercury compounds cause toxicity in various organs following accidental and / or occupational exposures. Mercury can lead to toxicity in the kidney and liver. Accumulation of mercury in the kidney causes acute renal failure (Emanuelli et al., 1996 ▶;Tanaka-Kagawa et al., 1998 ▶). The liver plays an important role in biotransformation of mercury. However, after exposure to mercury compounds, mercury localizes in the liver tissue (El-Shenawy and Hassan, 2008 ▶;Sridhar et al., 2013 ▶). Mercury is also well known as haematotoxic (Durak et al., 2010 ▶), neurotoxic (Moraes-Silva et al., 2014 ▶), and genotoxic chemical (Rozgaj et al., 2005 ▶) and exerts negative effects on the reproductive system in male rats (Kalender et al., 2013 ▶). Another target for mercury is thiol-containing enzymes (Emanuelli et al., 1996 ▶;Nogueira et al., 2003 ▶). Mercury binds to sulfhydryl groups and causes reduction of glutathione levels, leading to increases in the levels of reactive oxygen species (ROS), such as superoxide anion radicals, hydrogen peroxide and hydroxyl radicals (Stohs and Bagchi, 1995 ▶). Increased levels of ROS cause lipid, protein and DNA oxidation (Clarkson, 1997 ▶). Thus, since oxidative stress and endogenous thiol depletion play important roles in mercury-induced toxicity, antioxidants can be useful in treatment of mercury poisoning (Patrick, 2002 ▶;Pillai and Gupta, 2005 ▶). Recent studies have shown that melatonin (Nava et al., 2000 ▶; Şener et al., 2003 ▶), curcumin (Agarwal et al., 2010a ▶) and vitamin E (Agarwal et al., 2010b ▶) have protective effects against mercuric chloride (HgCl_2_)-induced acute renal toxicity. Also, Boroushaki et al., showed that pomegranate seed oil decreased HgCl_2_- toxicity in the kidney and liver (Boroushaki et al., 2016 ▶;Boroushaki et al., 2014b ▶). *Rheum turkestanicum* (Polygonaceae) is a plant that grows widely in central Asia and in Northeast of Iran. In traditional medicine, the root of *R. turkestanicum* has been used as an anti-diabetic, anti-hypertensive and anticancer agent (Dorsey and Kao, 2007 ▶). Rheum species contain antioxidant compounds. Rhapontigenin and rhaponticin isolated from *R. undulatum,* scavenge ROS, 1, 1-diphenyl-2-picrylhydrazyl (DPPH) radical, and hydrogen peroxide (H_2_O_2_) (Zhang et al., 2007 ▶). Also, these compounds decrease membrane lipid peroxidation and cellular DNA damage (Zhang et al., 2007 ▶). A recent study showed that some of antioxidant compounds isolated from *R. emodi* protect H9c2 cells against H_2_O_2_ –induced toxicity (Chai et al., 2012 ▶). In another study, it has been shown that *R. turkestanicum* reduces doxorubicin toxicity in H9c2 cell line via reduction of ROS production (Hosseini and Rajabian, 2016 ▶). Also, it was shown that *R. turkestanicum* reduced lipid peroxidation and oxidative stress in diabetic rats (Hosseini et al., 2017 ▶). In this research, the protective effect of root extract of *R. turkestanicum* was evaluated against mercuric chloride-induced nephrotoxicity and hepatotoxicity in rats.

## Materials and Methods

**Animals**

Adult male Wistar rats (obtained from Animal House, Faculty of Medicine, Mashhad University of Medical Sciences, Mashhad, Iran), weighing 220-250 g, were used in this research. Animals were housed in pathogen-free cages with 12 hr/ 12 hr light/dark cycles and they had with free access to food and water *ad libitum*. All procedures were approved by the University Ethics Committee and were performed in compliance with National Laws and National Institutes of Health guidelines for the use and care of laboratory animals.

**Chemicals**

DTNB (2,20-dinitro-5,50-dithiodibenzoic acid), TBA (2-thiobarbituric acid), n-butanol, Na_2_EDTA (ethylenediaminetetraacetic acid disodium salt), Trizma base [Tris (hydroxymethyl) aminomethane], HCl (hydrochloric acid), KCl (potassium chloride), phosphoric acid (1%), ether, TCA (Trichloroacetic acid) and methanol were purchased from Merck (Darmstadt, Germany). Mercuric chloride (HgCl2) was obtained from May & Baker (London, England). 

**Preparation of plant’s extract**

The root of *R. turkestanicum* Janisch. was collected from Chenar, a village in Zavin Rural District, Kalat County, Khorasan Razavi Province, Iran. The plant was identified by M.R. Joharchi, from Ferdowsi University, Mashhad, Iran and a voucher specimen of this plant was deposited (No. 21377). Dried roots were grounded to a fine powder and then, 50 g of this powder was subjected to extraction with 70% ethanol in a Soxhlet apparatus for 48 hr. The hydro-alcoholic extract was then dried on a water bath and stored in -18^◦^C freezer. The yield of extract was 21% (w/w). 

**Experimental design**

After acclimatization, animals were randomly divided into five groups (six rats in each group) and individually put in the metabolic cages. Group I (control) was treated with saline (1ml/kg). Group II received 200 mg/kg extract. Group III was treated with HgCl_2_ (5 mg/kg). Groups IV and V were treated with extract (100 and 200 mg/kg, respectively), 1 hr before receiving HgCl_2_. All procedures were done between 10–12 am. All treatments were given intraperitoneally for three days on a daily basis. On day 4, 24-hr urine samples were collected for measuring urinary glucose and protein concentrations. Twenty four hours after the last injection of HgCl_2_, all rats were anesthetized by ether. Blood samples were collected by cardiac puncture, and centrifuged at 1000 "g" for 15 min to separate the serum for assessment of biochemical parameters. The right kidney and liver were removed, homogenized in cold KCl solution (1.5%, pH=7) to give a 10% homogenate suspension and used for biochemical assays. A piece of the liver and the left kidney were fixed in 10% formalin and sectioned for histopathological studies.

**Biochemical methods**

Glucose concentration was assayed by an enzymatic method (glucose oxidase) and protein concentration was measured by a turbidimetric method (Lott and Turner, 1975 ▶;McElderry et al., 1982 ▶). Urea concentration was determined colorimetrically, using Autoanalyzer (Technicon RA-1000, London, England) and urea kit (Man Lab Company, Tehran, Iran). Creatinine concentration was measured by the Jaffe’s method (Masson et al., 1981 ▶). ALT and AST level measurement were done according to the International Federation of Clinical Chemistry (IFCC) method and expressed as units per liter (Adeneye and Olagunju, 2008 ▶).

**Calculation of MDA level**

Lipid peroxidation in the kidney tissues was measured based on the levels of malondialdehyde (MDA), which is the end-product of lipid peroxidation and reacts with TBA as a thiobarbituric acid reactive substance (TBARS) to produce a red-colored complex which has a peak absorbance at 532 nm (Hosseinzadeh et al., 2005 ▶). Briefly, 3 ml phosphoric acid (1%) and 1ml TBA (0.6%) were added to 0.5 ml of homogenate in a centrifuge tube and the mixture was heated for 45 min in a boiling water bath. After cooling, 4 ml n-butanol was added to the mixture, vortexed for 1 min, and centrifuged at 20,000 "g" for 20 min. The organic layer was transferred to a fresh tube and its absorbance was measured at 532 nm. 

MDA (mmol/g tissue) =absorbance/1.56×105

**Calculation of total thiol content**

Total SH groups were measured using DTNB. This reagent reacts with SH groups to produce a yellow colored complex which has a peak absorbance at 412 nm. Here, 1 ml Tris–EDTA buffer (pH=8.6) was added to 0.5 ml kidney homogenate in 2-ml cuvettes and absorbance was read at 412 nm against Tris–EDTA buffer alone (A1). Then, 20 µl DTNB reagent (10mM in methanol) was added to the mixture, and after 15 min (at room temperature), the sample absorbance was read again (A2). The absorbance of DTNB reagent alone was also read and recorded as blank (B). Total thiol concentration (mM) was calculated using the following equation (Boroushaki et al., 2014a ▶;Bouroshaki et al., 2010 ▶):

Thiol concentration (mM) = (A2-A1–B) ×1.07/0:05×13.6

**Histological study**

Liver and kidney tissue samples were fixed in 10% formalin for at least 24 hr. The fixed specimens were processed, using paraffin-embedding technique. Then, hematoxylin and eosin (H&E) staining was performed for histopathological examinations which were done under light microscopy (Oda and El-Ashmawy, 2012 ▶). 

**Statistical analysis**

Data were expressed as mean±SEM. Statistical analysis was performed using one-way analysis of variance (ANOVA) followed by Tukey–Kramer *post hoc* test for multiple comparisons. The p-values less than 0.05 were considered statistically significant.

## Results

**Biochemical studies**

*Serum AST and ALT measurement*

**Figure 1:**
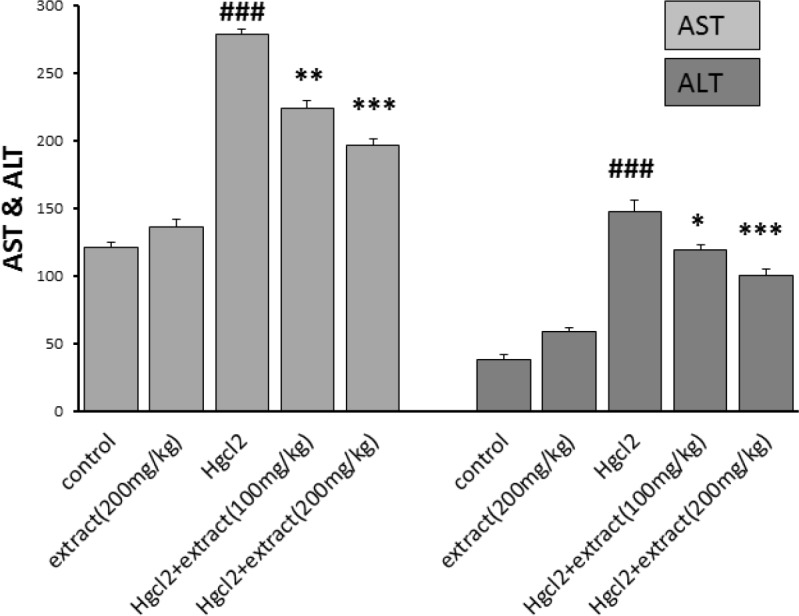
Effect of pre-treatment with *R. turkestanicum* against HgCl_2_ intoxication in terms of serum AST and ALT activity. Values are expressed as mean±SEM (n=6). ###p<0.001 as compared with control group. *p<0.05, **p<0.01 and ***p<0.001 as compared to HgCl_2_-treated group

ALT and AST were measured in serum. Results showed that, HgCl_2_ significantly increases ALT and AST (p<0.001). The extract at doses of 100 mg/kg (p<0.01) and 200mg/kg (p<0.001) decreased AST level in HgCl_2_-treated groups. Also, the extract at doses of 100mg/kg (p<0.05) and 200 mg/kg (p<0.01) decreased ALT levels. The levels of ALT and AST were not significantly different between control and extract-treated groups (Figure 1).

*Serum urea and creatinine measurement*

**Figure 2:**
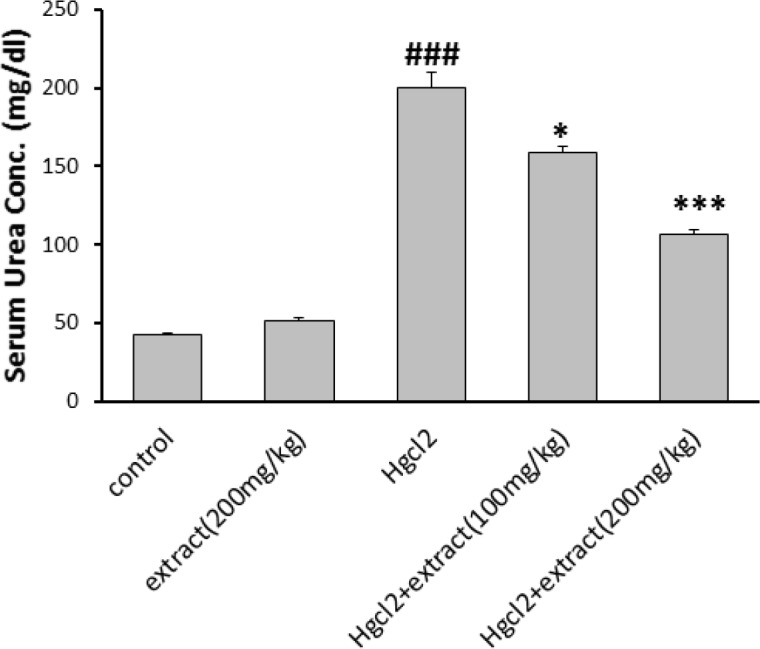
Effect of the pre-treatment with* R. turkestanicum* against HgCl_2_- intoxication in terms of serum urea levels. Values are expressed as mean±SEM (n=6). ### p<0.001 as compared to control group. *p<0.05 and ***p<0.001 as compared to HgCl_2_-treated group

**Figure 3:**
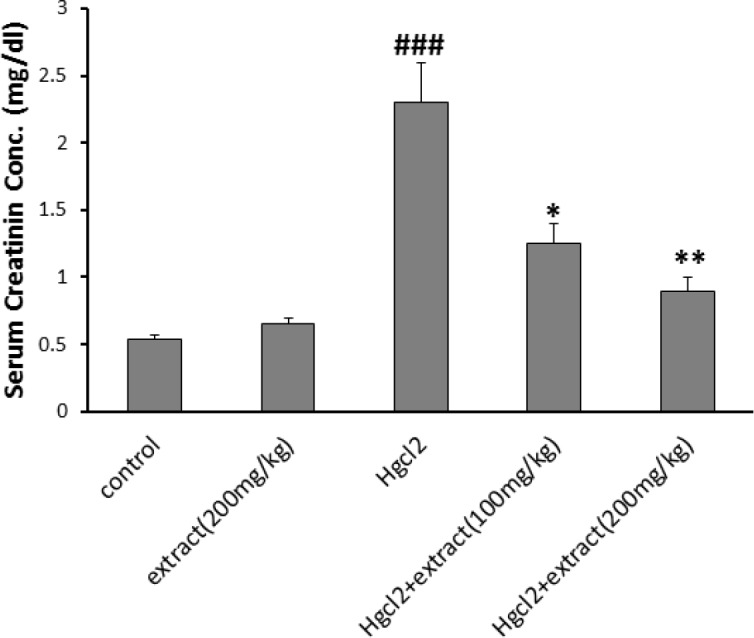
Effect of pre-treatment with *R. turkestanicum* against HgCl_2_- intoxication in terms of serum creatinine levels. Values are expressed as mean±SEM (n=6). ### p<0.001 as compared to control group. *p<0.05 and **p<0.01 as compared to HgCl_2_-treated group

The level of urea and creatinine increased in HgCl_2_ group (p<0.001). In groups 4 and 5, the extract decreased urea and creatinin at doses of 100 (p<0.05) and 200 mg/kg (p<0.001). The level of urea and creatinine in groups 2 and control were the same (Figures 2 and 3).

*Lipid peroxidation*

**Figure 4:**
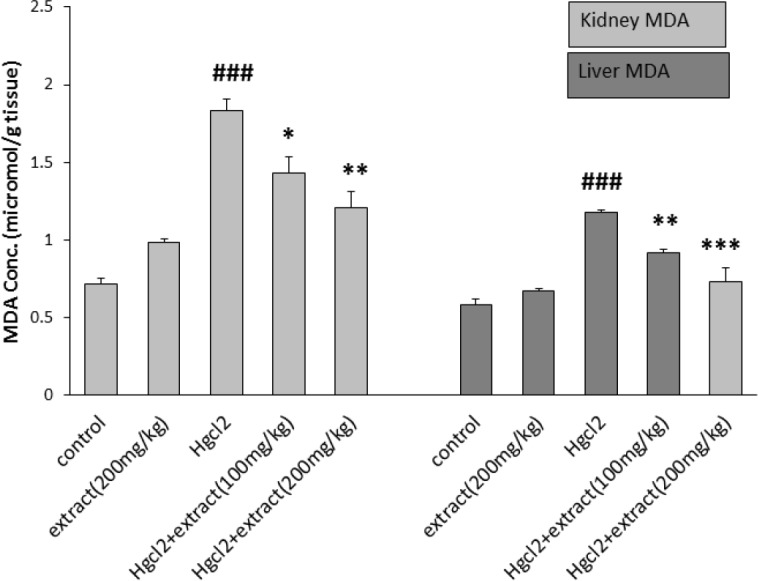
Effect of pretreatment with *R. turkestanicum* against HgCl_2_ intoxication in terms of MDA content in rats kidney and liver. Values are expressed as mean±SEM (n=6). ### p<0.001 as compared to control group. *p<0.05, **p<0.01 and ***p<0.001 as compared to HgCl_2_-treated group

The lipid peroxidation level in the kidney and liver was evaluated as malondialdehyde level (MDA). As shown in Figure 4, mercuric chloride administration, significantly increased MDA levels in the kidney compared to control group and group 2 (p<0.001). In groups 4 and 5, the extract at the doses of 100 mg/kg (p<0.05) and 200 mg/kg (p<0.01) significantly decreased renal MDA levels compared to HgCl_2_-treated group. In the liver, MDA levels were significantly increased in HgCl_2_-treated group compared to the group 2 and control group (p<0.001). In groups 4 and 5, MDA levels were decreased (p<0.01 for 100mg/kg and p<0.001 for 200mg/kg,). 

*Urinary glucose and protein measurement*

As shown in Table 1, HgCl_2_ increased the urinary levels of protein and glucose. In groups 4 and 5, the extract at the dose of 200 mg/kg decreased these parameters in comparison to HgCl_2_- treated group.

**Table 1:** Effect of *R. turkestanicum* on urinary levels of protein and glucose. Data are shown as mean±SEM (n=6)

	**Control** **saline (1ml/kg) **	**Extract ** **(200mg/kg)**	**HgCl**_2_ **(5mg/kg)**	**Extract (100mg/kg)+HgCl**_2_	**Extract (200mg/kg)+HgCl**_2_
**urinary protein (mg/dl)**	400±12	410±10.5	810±20 ###	640±27	595±19 *
**urinary glucose (mg/dl)**	40±5	35±3.7	746±21 ###	728±18	610±12 *

* p<0.05 as compared to HgCl_2_-treated animals and

### p<0.001 in comparison to control group.

*Total thiol content*

**Figure 5:**
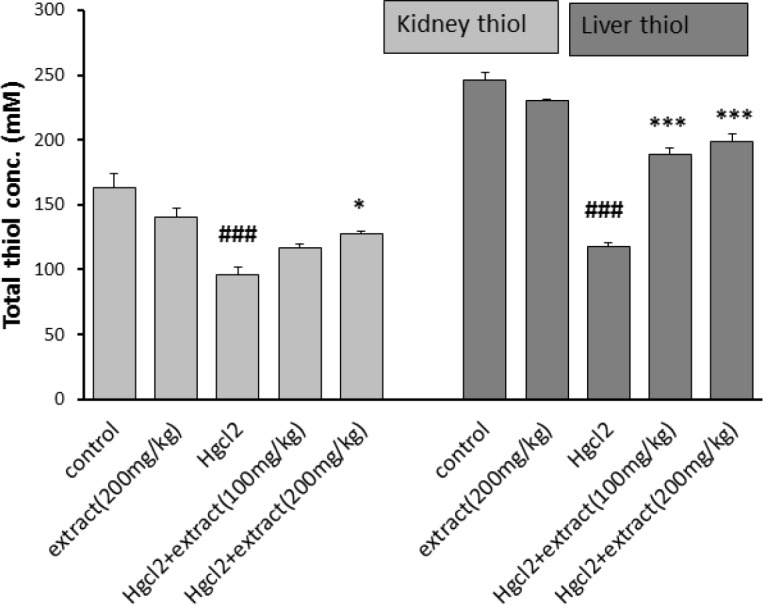
Effect of pretreatment with *R. turkestanicum* against HgCl_2_ intoxication in terms of thiol content in rats kidney and liver. Values are expressed as mean±SEM (n=6). ### p<0.001 as compared to control group. *p<0.05 and ***p<0.01 as compared to HgCl_2_-treated group

Figure 5 shows that HgCl_2_ significantly decreased the total thiol content in the liver and kidney homogenates (p<0.001) compared to the control group and group 2. In groups 4 and 5, the extract increased thiol content in kidney tissue only at the dose of 200 mg/kg (p<0.05), but increased the thiol content in the liver at both doses (p<0.001), compared to the group 3. 

**Histopathological Observations**

**Figure 6:**
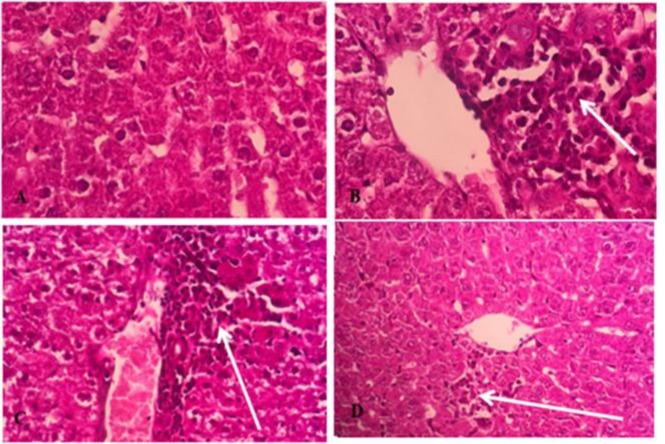
Photomicrographs showing the pathological changes of liver tissues after HgCl_2_ exposure and pretreatment with* R.turkestanicum*. A) Control: normal liver tissue (X100); B) Group treated with 5 mg/kg HgCl_2_ showing inflammatory cells infiltration and Peri central vein inflammation (2+) (X100); C) Rats treated with extract 100 mg/kg of extract showed portal inflammation (2+) (X100) and D) Rats treated with extract 200 mg/kg showed peri central vein inflammation (1+) (X100). Liver injuries were reduced in extract-treated groups. The (+) sign shows the severity of injury as 1+ indicates mild and 2+ indicates moderate damages

**Figure 7:**
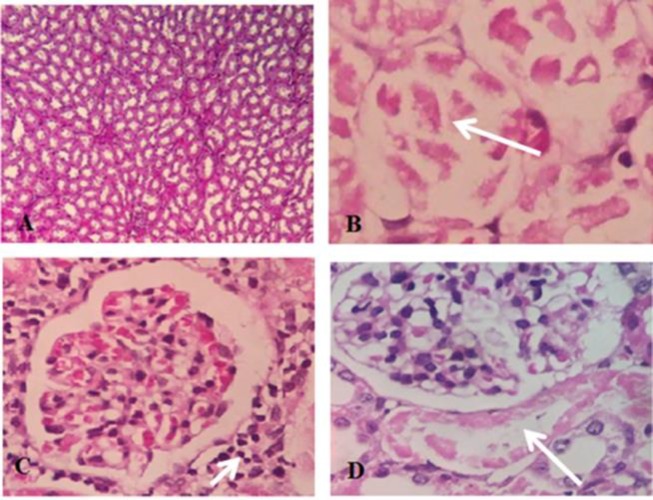
Photomicrographs showing the pathological changes of kidney tissues after HgCl_2_ exposure and pretreatment with* R.turkestanicum*. A) Control: normal kidney tissue (X×100); B) Group treated with 5 mg/kg HgCl2 showing tubular necrosis (3+) (X 400); C) Rats treated with extract 100 mg/kg +HgCl_2_ showed peri glomerular inflammation (2+) (X 400) and D) Rats treated with extract 200 mg/kg+ HgCl_2_ showed focal necrosis of proximal tubule (1+) (X400). Lower levels of kidney injuries were observed in extract-treated groups. The (+) sign shows the severity of injery.1+ (mild), 2+ (moderate) and 3+ (severe)

Liver histopathological studies showed inflammatory, nfiltration around the tubular central vein in HgCl_2_-treated group (Figure 6). Liver sections pre-treated with extract (200 mg/kg) showed partial recovery and lower infiltration compared to HgCl_2_ group (Figure 6). Renal histopathological changes are shown in Figure 7. There was no evidence of structural changes nor tissue damage in control group. In HgCl_2_-treated group, severe tubular necrosis and atrophy of renal tissue were observed. Structural and morphological changes in the extract- treated groups were lower compared to HgCl_2_ group (Figure 7) 

## Discussion

Our study demonstrated that treatment of rats with HgCl_2_ significantly enhances MDA level which is in agreement with the results of previous reports (Agarwal et al., 2010b ▶;Miller et al., 1991 ▶). Recent studies have shown that mercuric chloride increases the production of many ROS such as superoxide and H_2_O_2_ (Huang et al., 1996 ▶), which cause lipid peroxidation and subsequently oxidative tissue damage (Gstraunthaler et al., 1983 ▶; Linden et al., 2008 ▶). Mercuric chloride has a great affinity for thiol groups (–SH) of endogenous biomolecules (Clarkson, 1997 ▶). Some proteins and agents of low molecular weight such as cysteine and glutathione (GSH) which are found in body, contain thiol group and are regarded as a target for mercury. Therefore, mercury inhibits the activities of antioxidant enzymes such as GSH, via binding to thiol groups (Li et al., 2011 ▶; Zalups, 2000 ▶). Our study showed that mercuric chloride decreased the thiol content in liver and kidney tissues while the extract increased the thiol level in tissues. Also, in this research, the activity of serum transaminases (AST and ALT) increased after HgCl_2_ administration which was is agreement with another study (El-Shenawy and Hassan, 2008 ▶). Elevation of these enzymes is related to hepatocellular necrosis which led to release of these enzymes into the blood (Sharma et al., 2007 ▶). These enzymes reduced significantly in extract-treated groups in comparison to HgCl_2_-group. Also, histological changes of kidney revealed that pretreatment with *R. turkestanicum* resulted in a significant and dose-dependent decrease in the rate of tubular atrophy and necrosis. In group 4, which received extract 200mg/kg, mercury toxic effects in terms of necrosis and cellular casts were reduced compared to groups 2 and 3. Histological changes in the liver showed inflammatory, infiltration in HgCl_2_-treated group while these changes decreased in extract-treated groups especially those treated with the higher dose of extract. Recent studies have shown that most herbal medicines improve tissue damages by their antioxidant activities (Alam et al., 2005 ▶;Van Acker et al., 1996 ▶). Phenols and polyphenolic compounds, such as flavonoids, are widely found in food products derived from plant sources, and they have been shown to possess significant antioxidant activities (Andiç et al., 2009 ▶;Ebrahimzadeh and Bahramian, 2009 ▶). In the present study, we showed that *R. turkestanicum* root extract has a potent antioxidant activity, and could protect the kidney and liver against HgCl_2_- induced toxicity via reduction of MDA and increasing of thiol levels. The main bioactive components of rheum species are anthraquinone derivatives including emodin, aloe-emodin, rhein, chrysophanol, physcion, and danthron. Other constituents such as dianthrones, stilbenes, anthocynins, falvonoids, anthraglycosides, polyphenols, essential oil, organic acids and chromone glycosides (Alam et al., 2005 ▶). Due to the presence of anti-oxidant compounds in rheum species, probably this genus has protective effects against oxidative stress. A study showed that *R. emodi* has protective effects against cadmium chloride and mercuric chloride toxicity (Alam et al., 2005 ▶). Also, emodin ameliorates cisplatin-induced apoptosis in rats renal tubular cells *in vitro* through modulating the AMPK/mTOR signaling pathways and activating autophagy (Zhen-kui et al., 2014 ▶). Other studies showed that emodin can alleviate ischemia/reperfusion injury in renal transplantation, possibly through reduction of lipid peroxidation and inhibition of inflammatory factors production (Liu et al., 2016 ▶). *R. ribes *root improved renal dysfunction in alloxan-induced diabetic rats by controlling blood glucose and exerting protective effects in the kidneys (Hamzeh et al., 2014 ▶). Therefore, protective effects of *R. turkestanicum* may be related to the anti-oxidant activity of its active compounds. More studies are needed to provide a better understanding of protective effects* R. turkestanicum *root extract. 

This study revealed that *R. turkestanicum* extract protects the liver and kidney against HgCl_2_-induced toxicity. This protective effect may be related to anti-oxidant activity of the extract. More investigations are needed to discover the underlying exact mechanism(s). 
